# Effects of Exposure Period on the Developmental Toxicity of 2-Bromopropane in Sprague-Dawley Rats

**DOI:** 10.5487/TR.2008.24.4.263

**Published:** 2008-12-01

**Authors:** In-Sik Shin, Jong-Chan Lee, Kang-Hyeon Kim, Tai-Hwan Ahn, Chun-Sik Bae, Changjong Moon, Sung-Ho Kim, Dong-Ho Shin, Jong-Choon Kim

**Affiliations:** Animal Medical Center, College of Veterinary Medicine, Chonnam National University, Gwangju, 500-757 Korea

**Keywords:** 2-Bromopropane, Embryotoxicity, Teratogenicity, Susceptible period, Metabolic activation, Rats

## Abstract

Recently we reported that 2-bromopropane (2-BP) has maternal toxicity, embryotoxicity, and teratogenicity in Sprague-Dawley rats. The aims of this study are to examine the potential effects of 2-BP administration on pregnant dams and embryo-fetal development, and to investigate the effects of metabolic activation induced by phenobarbital (PB) on developmental toxicities of 2-BP. Pregnant rats received 1000 mg/kg/day subcutaneous 2-BP injections on gestational days (GD) 6 through 10 (Group II and Group IIII) or 11 through 15 (Group IV). Pregnant rats in Group III received an intraperitoneal PB injection once daily at 80 mg/kg/day on GD 3 through 5 for induction of the liver metabolic enzyme system. Control rats received vehicle injections only on GD 6 through 15. All dams underwent caesarean sections on GD 20 and their fetuses were examined for external, visceral, and skeletal abnormalities. Significant adverse effects on pregnant dams and embryo-fetal development were observed in all the treatment groups, and the maternal and embryo-fetal effects of 2-BP observed in Group II were higher than those seen in Group IV. Conversely, maternal and embryo-fetal developmental toxicities observed in Group III were comparable to those seen in Group II. These results suggest that the potential effects of 2-BP on pregnant dams and embryo-fetal development are more likely in the first half of organogenesis (days 6~10 of pregnancy) than in the second half and that the metabolic activation induced by PB pre-treatment did not modify the developmental toxic effects of 2-BP in rats.

## INTRODUCTION

2-Bromopropane (2-BP, CAS No. 75-26-3) is a halogenated propane analogue and is also known as isopropyl bromide. It is a colorless and nonflammable liquid with a melting point -90.0°C, boiling point of 59.4°C and a solubility of 3.2 mg/ml in water. It may function as a substitute for chlorofluorocarbons which contribute to destruction of the ozone layer and global warming.

Several Korean researchers have reported that a cluster of patients with amenorrhea and oligozoospermia had occupational exposure to solvents containing 2-BP in 1995 (Kim *et al.*, 1996). Epidemiological studies have suggested that 2-BP may have acted as the causative agent of these health disorders (Kim *et al.*, 1996; Park *et al.*, 1997). Multiple studies have reported that 2-BP is well known reproductive, hematopoietic, central nervous, and immune system toxicant (Ichihara *et al.*, 1997; Lee *et al.*, 1998; Omura *et al.*, 1999; Son *et al.*, 1999; Wu *et al.*, 2002; Yu *et al.*, 1999; Zhao *et al.*, 2002). Several studies have additionally revealed that 2-BP is a DNA-damaging agent, and that the DNA damage induced by 2-BP might be related to 2-BP toxicities (Wu *et al.*, 2002; Zhao *et al.*, 2002). Maeng and Yu (1997) suggested that a dose-related increase in mutations was noted with 2-BP treatment in *Salmonella typhimurium* strain TA 100 with S9 activation, and this suggested that metabolic enzyme induction by the S9 fraction could modify 2-BP's toxic potential. A reproductive and developmental toxicity study of 2-BP reported that administration of multiple subcutaneous 2-BP injections to pregnant and lactating rats caused increased peri- and post-natal deaths, suppressed body weight and development, and increased incidence of reproductive organ dysfunction of F1 offspring at dose levels of 405 mg/kg/day or greater (Kang *et al*., 2002). We have recently demonstrated that multiple subcutaneous 2-BP injections to pregnant rats and mice resulted in severe developmental toxicities including teratogenicity at the dose levels of 1000 mg/kg/day (Kim *et al*., 2003, 2004). However, we did not confirm the susceptible phase of developmental toxicity of 2-BP in gestation because of the timing of injections. Further, the studies did not investigate the potential effects of metabolic enzyme induction on 2-BP's developmental toxicity in rats.

Phenobarbital (PB) is the oldest and most widely used anticonvulsant worldwide. It has sedative and hypnotic properties. Rouer *et al*. (1982) and Sunouchi *et al*. (1984) reported that PB induced drug metabolic enzymes in the liver and was particularly effective for the induction of the cytochrome P450 of liver metabolic enzymes. The metabolic enzymes induced by PB enhanced drug hydroxylation and glucuronidation (Vainio *et al*., 1974), and metabolic enzyme induction can profoundly alter the toxicologic properties of chemicals.

This study was conducted to determine a phase specificity of the development toxicity of 2-BP and to confirm the effects of liver metabolism on this toxicity in Sprague-Dawley rats. The phase specificity of 2-BP was anticipated to yield valuable information about the teratogenesis and the developmental toxicity of 2-BP.

## MATERIALS AND METHODS

***Animals.*** Male and nulliparous female Sprague-Dawley rats were purchased from Bio Genomics Inc. (Seoul, Korea) and used after one week of quarantine and acclimatization. Rats were 10 weeks old and were maintained at a temperature of 23 ± 3°C, a relative humidity of 50 ± 10%, and under a controlled 12-h light/dark cycle. Only healthy animals were assigned to the study. Mating was conducted by placing 2 females and 1 one male in a cage overnight, and successful mating was confirmed by the presence of sperm in the vaginal smear. The first 24 h after mating was designated day 0 of gestation (GD 0). Mated females were housed individually in clear polycarbonate cages with stainless-steel wire lids, were given tap water sterilized by ultraviolet irradiation, and fed a commercial rodent chow (Samyang Feed Co, Wonju, Korea) ad libitum. The Institutional Animal Care and Use Committee of Chonnam National University approved the protocols for the animal study, and the animals were cared for in accordance with the Guidelines for Animal Experiments of Chonnam National University.

***Test chemical and treatment.*** 2-BP was purchased from Aldrich (Milwaukee, WI, USA) and PB was supplied by the Daehan Pharmaceutical Company (Seoul, Korea). The 2-BP was dissolved in corn oil (Sigma, St. Louis, MO, USA) and the PB was dissolved in normal saline, and both were freshly prepared on a daily basis prior to treatment. The daily application volumes (5 ml/kg body weight) of 2-BP and PB were calculated in advance based on body weight. The subcutaneous 2-BP injections were administered to pregnant rates from either GD 6 through GD 10 or from GD 11 through GD 15. Control rats received an equivalent volume of corn oil alone from GD 6 through GD15. Metabolic enzyme induction was initiated by intraperitoneal PB injections from GD 3 through GD 5. The toxic dose (1000 mg/kg/day) of 2-BP used to elicit developmental abnormalities in this study was selected based on results from our previous study, which suggested that multiple subcutaneous 2-BP doses in the organogenetic period were embryotoxic and teratogenic at 1000 mg/kg/day (Kim *et al*., 2004). The PB dose was selected as 80 mg/kg/day, which is well documented to induce hepatic microsomal enzyme in rats (Chung *et al*., 1997; Pappas *et al*., 2001). Although the major exposure routes of 2-BP in human are dermal or inhalation, the subcutaneous route was selected by reasons that a greater amount of the chemical can be dosed by the subcutaneous route rather than the routes, that the subcutaneous dose provides accuracy in estimating the amount of test chemical taken into the organism, and that the absorption route of subcutaneous dose may be similar to that of dermal dose.

***Experimental groups.*** Inseminated female rats (n = 32) were randomly assigned to 4 experimental groups of 8 rats each: Group I (control) received the vehicles only, Group II received 2-BP (1000 mg/kg/day) from GD 6 through GD 10, group III received PB (80 mg/kg/day) from GD 3 through GD 5 and 2-BP (1000 mg/kg/day) from GD 6 through GD 10, and Group IV received 2-BP (1000 mg/kg/day) from GD 11 through GD 15.

***Observation of dams.*** Clinical observation of all pregnant females was made daily throughout gestation and abnormal signs were individually recorded for type, observation day, observation time, and duration. Maternal body weights were measured on GD 0, 6, 11, 16, and 20. Individual food consumption was determined on GD 0, 6, 11, 16, and 19. Pregnant females were sacrificed on GD 20 and the main viscera were observed by macroscopic examination.

***Caesarean section.*** The ovaries and uterus were removed from each female and examined for organ weight, the number of corpora lutea, and the status of all implantation sites to examine fetal viability, early and late resorptions, and total implantations. Resorption was classified as early when only placental tissue was visible and late when placental and embryonic tissue were visible at caesarean section. All live fetuses were sexed, weighed, and inspected for external morphological abnormalities including cleft palates. Alternate fetuses were selected for either skeletal or visceral examination. Skeletal evaluations of 5% formalin-fixed fetuses were performed after staining skeletons with Alizarin Red S and clearing the stains with potassium hydroxide solution by a modified Dawson's method (1926). The visceral examination of Bouin's fluid-fixed fetuses was performed with a freehand razor sectioning technique (Wilson, 1965) for the head and abdomen and Nishimura's method (1974) for the thorax. External, visceral, and skeletal findings were classified as developmental malformations, variations, or retardations, respectively. Specific terminology was used classify the structural developmental abnormalities in common laboratory mammals as previously described (Wise *et al.*, 1997).

***Statistical analyses.*** The unit for statistical measurement was the pregnant female or the litter (Weil, 1970). Statistical analyses of experimental and control values were performed using the Student's t-test. The gender ratio and the proportion of the litters with malformations and developmental variations were compared using a chi-squared test and the Fisher's exact probability test (Fisher, 1970). The statistical analyses were performed using the GraphPad InStat software (GraphPad Software Inc. San Diego, CA). The significance of the differences between the groups was estimated at the probability levels of 1 and 5%.

## RESULTS

***Maternal toxicity.*** The clinical findings for the 2-BP-treated pregnant rats are summarized in Table 1. All females survived in both control and treatment groups throughout the study. The treatment-related clinical signs including reddish tears from the eyes, nasal discharge, fur staining, weakness, piloerection, and swelling at the subcutaneous injection sites. These signs were observed in all treatment groups, and the incidence and severity of the clinical signs were similar among the treatment groups.

Table 2 describes the changes in body weight in each group during the study period. The maternal body weights on GD 20 in Group II, from GD 16 through 20 in Group III, and on GD 20 in Group IV were significantly reduced when compared with the control groups. No significant differences were noted between treat-

**Table 1.** Clinical findings of pregnant rats treated with 2-bromopropane during the organogenetic period

| Items | Groups | | | |
|---|---|---|---|---|
| | I | II | III | IV |
| Number of mated females | 8 | 8 | 8 | 8 |
| Number of pregnant females | 8 | 7 | 7 | 7 |
| Number of dams with clinical signs | 0 | 3 | 4 | 2 |
| Reddish tear | 0 | 0 | 2 | 1 |
| Nasal discharge | 0 | 0 | 0 | 2 |
| Fur staining | 0 | 2 | 3 | 2 |
| Weakness | 0 | 0 | 1 | 0 |
| Piloerection | 0 | 1 | 0 | 0 |
| Ataxia | 0 | 0 | 1 | 0 |
| Swelling at the sc injection sites | 0 | 2 | 1 | 1 |

**Note:** Group I (control) received the respective vehicles only; Group II received 2-BP (1000 mg/kg/day) from GD 6 through 10; group III received PB (80 mg/kg/day) from GD 3 through 5 and 2-BP (1000 mg/kg/day) from GD 6 through 10; and Group IV received 2-BP (1000 mg/kg/day) from GD 11 through 15.

**Table 2.** Body weights (g) of pregnant rats treated with 2-bromopropane during the organogenetic period

| Parameters | Groups | | | |
|---|---|---|---|---|
| | I | II | III | IV |
| Number of dams | 8 | 7 | 7 | 7 |
| Gestational day 0 | 220.6 ± 16.32[a] | 223.9 ± 8.97 | 219.4 ± 18.59 | 214.7 ± 11.13 |
| Gestational day 6 | 252.5 ± 17.18 | 254.3 ± 10.42 | 232.4 ± 21.98 | 241.1 ± 17.79 |
| Gestational day 11 | 295.9 ± 21.03 | 286.9 ± 12.27 | 269.3 ± 22.05 | 273.0 ± 19.00 |
| Gestational day 16 | 336.4 ± 22.80 | 315.3 ± 17.70 | 299.7 ± 24.38* | 307.0 ± 19.03 |
| Gestational day 20 | 407.8 ± 28.44 | 343.6 ± 26.30** | 327.7 ± 30.35** | 349.7 ± 36.09** |

**Note:** Group I (control) received the respective vehicles only; Group II received 2-BP (1000 mg/kg/day) from GD 6 through 10; group III received PB (80 mg/kg/day) from GD 3 through 5 and 2-BP (1000 mg/kg/day) from GD 6 through 10; and Group IV received 2-BP (1000 mg/kg/day) from GD 11 through 15.

[a]Values are expressed as mean ± SD.

*, **Significant difference at $p < 0.05$ and $p < 0.01$ levels, respectively, when compared with Group I.

**Table 3.** Food consumption (g) of pregnant rats treated with 2-bromopropane during the organogenetic period

| Parameters | Groups | | | |
|---|---|---|---|---|
| | I | II | III | IV |
| Number of dams | 8 | 7 | 7 | 7 |
| Gestational day 0 | 22.3 ± 4.20[a] | 21.7 ± 3.64 | 19.6 ± 5.62 | 22.7 ± 6.90 |
| Gestational day 6 | 23.5 ± 3.92 | 20.4 ± 3.55 | 24.3 ± 6.39 | 19.3 ± 2.81 |
| Gestational day 11 | 24.9 ± 4.06 | 24.7 ± 2.36 | 25.7 ± 4.27 | 22.9 ± 3.09 |
| Gestational day 16 | 25.6 ± 4.63 | 23.7 ± 2.93 | 24.4 ± 5.82 | 26.1 ± 4.76 |
| Gestational day 20 | 27.8 ± 5.27 | 21.7 ± 4.39 | 18.7 ± 8.07 | 25.0 ± 6.89 |

Note: Group I (control) received the respective vehicles only; Group II received 2-BP (1000 mg/kg/day) from GD 6 through 10; group III received PB (80 mg/kg/day) from GD 3 through 5 and 2-BP (1000 mg/kg/day) from GD 6 through 10; and Group IV received 2-BP (1000 mg/kg/day) from GD 11 through 15.
[a]Values are expressed as mean ± SD.

ment groups and controls for food consumption during pregnancy (Table 3).

***Developmental toxicity.*** Table 4 summarizes the reproductive findings of the pregnant rats treated with 2-BP during the organogenetic period. The overall pregnancy rates were similar in all groups, ranging from 85 to 100%. No significant differences were observed in the number of corpora lutea, implantation, pre-implantation losses, and sex ratios of live fetuses in the treatment groups compared with control groups. However, the gravid uterine weights in Group II and Group III and litter sizes and placental weights in Group II were significantly lower than in the control groups. A dam in Group III (1/7) had complete resorption of implanted embryos at the scheduled caesarean section on GD 20. The number of fetal deaths and post implantation losses in Groups II and III were significantly higher when compared with the control group. Conversely, gravid uterine and placental weights, fetal deaths, and post-implanta-

**Table 4.** Caesarean section data of pregnant rats treated with 2-bromopropane during the organogenetic period

| Parameters | Groups | | | |
|---|---|---|---|---|
| | I | II | III | IV |
| Number of mated females | 8 | 8 | 8 | 8 |
| Number of pregnant females | 8 | 7 | 7 | 7 |
| Gravid uterine weight | 87.2 ± 16.72 | 39.2 ± 21.79** | 43.6 ± 29.36** | 66.0 ± 23.64# |
| Number of females totally resorbed | 0 | 0 | 1 | 0 |
| Number of corpora lutea | 15.6 ± 1.85[a] | 15.3 ± 1.38 | 16.3 ± 2.69 | 15.4 ± 2.76 |
| Number of implantations | 14.3 ± 1.16 | 13.1 ± 2.48 | 14.1 ± 2.61 | 12.1 ± 5.93 |
| Pre-implantation loss (%)[b] | 7.7 ± 6.97 | 14.6 ± 9.20 | 12.9 ± 9.71 | 24.9 ± 32.32 |
| Fetal deaths | 0.6 ± 0.74 | 5.7 ± 3.50* | 6.4 ± 6.55* | 1.0 ± 1.55## |
| Resorptions: Early | 0.6 ± 0.74 | 5.7 ± 3.50* | 6.0 ± 6.03* | 0.9 ± 1.21## |
| Late | 0 | 0 | 0.4 ± 0.79 | 0.1 ± 0.38 |
| Dead fetuses | 0 | 0 | 0 | 0 |
| Post-implantation loss (%)[c] | 4.4 ± 5.10 | 46.7 ± 29.88** | 42.1 ± 35.49** | 6.2 ± 7.03## |
| Litter size | 13.6 ± 1.30 | 7.4 ± 5.06* | 7.7 ± 4.89 | 11.1 ± 5.21 |
| Male/female | 50/59 | 28/24 | 22/32 | 34/4 |
| Sex ratio (male/female) | 0.85 | 1.17 | 0.69 | 0.77 |
| Fetal body weight (g) | | | | |
| Male | 4.0 ± 0.20 | 3.3 ± 0.94 | 3.4 ± 0.98 | 3.9 ± 0.28 |
| Female | 3.8 ± 0.19 | 3.2 ± 0.72 | 3.3 ± 0.88 | 3.1 ± 1.39 |
| Placental weight (g) | 0.5 ± 0.08 | 0.4 ± 0.06** | 0.5 ± 0.09 | 0.5 ± 0.08# |

Note: Group I (control) received the respective vehicles only; Group II received 2-BP (1000 mg/kg/day) from GD 6 through 10; group III received PB (80 mg/kg/day) from GD 3 through 5 and 2-BP (1000 mg/kg/day) from GD 6 through 10; and Group IV received 2-BP (1000 mg/kg/day) from GD 11 through 15.
[a]Values are expressed as mean ± SD.
[b]Pre-implantation loss (%) = [(no. of corpora lutea - no. of implantations)/no. of corpora lutea] × 100.
[c]Post-implantation loss (%) = [(no. of implantation sites - no. of live fetuses)/no. of implantation sites] × 100.
*, **Significant difference at $p < 0.05$ and $p < 0.01$ levels, respectively, when compared with Group I.
#, ##Significant difference at $p < 0.05$ and $p < 0.01$ levels, respectively, when compared with Group II.

**Table 5.** External alterations in fetuses of pregnant rats treated with 2-bromopropane during the organogenetic period

| Parameters | Groups | | | |
|---|---|---|---|---|
| | I | II | III | IV |
| Fetuses examined | 109 | 52 | 54 | 78 |
| Litters examined | 8 | 7 | 6 | 7 |
| Fetuses with malformation (%)[a] | 0 | 1 (1.9) | 1 (1.9) | 0 |
| Litters affected (%)[b] | 0 | 1 (14.3) | 1 (16.7) | 0 |
| Subcutaneous hemorrhage | 0 | 1 | 1 | 0 |
| Domed head | 0 | 1 | 0 | 0 |

Note: Group I (control) received the respective vehicles only; Group II received 2-BP (1000 mg/kg/day) from GD 6 through 10; group III received PB (80 mg/kg/day) from GD 3 through 5 and 2-BP (1000 mg/kg/day) from GD 6 through 10; and Group IV received 2-BP (1000 mg/kg/day) from GD 11 through 15.
[a]A single fetus may be represented more than once in listing individual defects.
[b]Includes litters with one or more affected fetuses.

tion losses in Group IV were significantly higher than those in Group II.

Table 5 summarizes the types and incidences of external abnormalities observed in the live fetuses. Although a few Group II and III fetuses displayed external anomalies (subcutaneous hemorrhage and domed head) there were no significantly differences compared with the control group. External anomalies appeared in 1 of 52 Group II fetuses (1.9%), in 1 of 7 Group II litters (14.3%), in 1 of 54 Group III fetuses (1.9%), and in 1 of 6 Group III litters (16.7%). No external anomalies were noted in Group IV fetuses.

Table 6 summarizes the types and incidences of visceral malformations and their variations observed in fetuses. The numbers of fetuses with visceral malformations in Group II and Group III were significantly higher than those in the control group. The litters with viscerally malformed fetuses in these groups were non-significantly higher than in controls. Visceral malformations included dilated lateral and third ventricles, the presence of a single ventricle, anophthalmia, microphthalmia, absent testis, and dilated cerebral ventricles. Such malformations were noted in 9 of 24 Group II fetuses (35.7%), in 5 of 7 Group II litters (71.4%), in 3 of 24 Group III fetuses (12.5%), and in 3 of 6 Group III litters (30.0%). No external malformations were noted in Group IV fetuses. Fetal numbers with visceral variations in Groups II and III were significantly higher when compared with the control values. The incidence of litters with affected fetuses in Groups II and III were also higher than in the control group, but these were non-

**Table 6.** Visceral abnormalities in fetuses of pregnant rats treated 2-bromopropane during the organogenetic period

| Parameters | Groups | | | |
|---|---|---|---|---|
| | I | II | III | IV |
| Fetuses examined | 52 | 24 | 24 | 36 |
| Litters examined | 8 | 7 | 6 | 7 |
| *Malformation* | | | | |
| Fetuses with malformation (%)[a] | 0 | 9 (37.5)** | 3 (12.5)* | 0## |
| Litters affected (%)[b] | 0 | 5 (71.4) | 3 (50) | 0 |
| Anophthalmia | 0 | 3 | 2 | 0 |
| Microphthalmia | 0 | 3 | 1 | 0 |
| Absent testis | 0 | 1 | 0 | 0 |
| Single ventricle | 0 | 2 | 0 | 0 |
| Dilated lateral & 3rd ventricle | 0 | 1 | 1 | 0 |
| *Variations* | | | | |
| Fetuses with variations (%) | 5 (9.6) | 13 (54.2)** | 9 (37.5)* | 7 (19.4) |
| Litters affected (%) | 2 (25) | 7 (100) | 6 (100) | 5 (71.4) |
| Misshapen thymus | 2 | 2 | 6 | 3 |
| Dilated renal pelvis | 1 | 2 | 1 | 2 |
| Dilated ureter | 2 | 8 | 3 | 3 |
| Misshapen heart | 0 | 2 | 0 | 0 |
| Dilated atrium | 0 | 1 | 0 | 0 |
| Convoluted ureter | 0 | 0 | 1 | 0 |

Note: Group I (control) received the respective vehicles only; Group II received 2-BP (1000 mg/kg/day) from GD 6 through 10; group III received PB (80 mg/kg/day) from GD 3 through 5 and 2-BP (1000 mg/kg/day) from GD 6 through 10; and Group IV received 2-BP (1000 mg/kg/day) from GD 11 through 15.
[a]A single fetus may be represented more than once in listing individual defects.
[b]Includes litters with one or more affected fetuses.
*, **Significant difference at $p < 0.05$ and $p < 0.01$ levels, respectively, when compared with Group I.
##Significant difference at $p < 0.01$ level compared with the Group II.

**Table 7.** Skeletal abnormalities in fetuses of pregnant rats treated 2-bromopropane during the organogenetic period

| Parameters | Groups | | | |
|---|---|---|---|---|
| | I | II | III | IV |
| Fetuses examined | 57 | 28 | 29 | 41 |
| Litters examined | 8 | 7 | 6 | 7 |
| *Malformation* | | | | |
| Fetuses with malformation (%)[a] | 0 | 4 (14.3)* | 2 (6.9) | 0# |
| Litters affected (%)[b] | 0 | 2 (28.6) | 2 (33.3) | 0 |
| Fused sternum | 0 | 2 | 1 | 0 |
| Cleaved sternum | 0 | 2 | 1 | 0 |
| Fused rib | 0 | 2 | 0 | 0 |
| Absent vertebra | 0 | 1 | 0 | 0 |
| *Variations* | | | | |
| Fetuses with variations (%) | 4 (7.0) | 10 (35.7)** | 9 (31.0)* | 10 (24.4)* |
| Litters affected (%) | 2 (37.5) | 7 (100) | 5 (85.3) | 4 (57.1) |
| Cervical rib | 0 | 2 | 1 | 0 |
| Supernumerary lumbar vertebra | 0 | 1 | 0 | 0 |
| Short supernumerary rib | 1 | 3 | 4 | 8 |
| Full supernumerary rib | 0 | 1 | 1 | 0 |
| Enlarged fontanel | 0 | 1 | 2 | 0 |
| Misshapen sternebra | 0 | 0 | 3 | 0 |
| *Retardations* | | | | |
| Fetuses with retardation (%) | 7 (12.3) | 24 (85.7)** | 21 (72.4)** | 20 (48.8)** |
| Litters affected | 4 (50) | 6 (85.7) | 6 (100) | 7 (100) |
| Incomplete ossification of supraoccipital | 1 | 3 | 5 | 0 |
| Dumbbell ossification of thoracic centrum | 6 | 23* | 18 | 20 |
| Unossified pulvis | 0 | 1 | 0 | 0 |
| Incomplete ossification of paretal | 0 | 1 | 0 | 0 |
| Incomplete ossification of interparietal | 0 | 0 | 1 | 0 |
| *Ossification degree* | | | | |
| Fetuses with ossification centers | | | | |
| Sternebra | 5.3 ± 0.27[c] | 3.3 ± 1.52** | 4.0 ± 1.52 | 5.6 ± 0.41## |
| Metacarpals in both forelimbs | 7.5 ± 0.62 | 2.9 ± 1.43** | 3.4 ± 0.44** | 4** |
| Metatarpals in hindlimbs | 8.0 ± 0.40 | 3.3 ± 1.46** | 3.6 ± 0.42** | 4** |
| Sacral & caudal vertebra | 9.3 ± 0.64 | 6.8 ± 2.20* | 7.2 ± 1.69 | 8.6 ± 0.85 |

Note: Group I (control) received the respective vehicles only; Group II received 2-BP (1000 mg/kg/day) from GD 6 through 10; group III received PB (80 mg/kg/day) from GD 3 through 5 and 2-BP (1000 mg/kg/day) from GD 6 through 10; and Group IV received 2-BP (1000 mg/kg/day) from GD 11 through 15.
[a]A single fetus may be represented more than once in listing individual defects.
[b]Includes litters with one or more affected fetuses.
[c]Values are expressed as mean ± SD.
*, **Significant difference at $p < 0.05$ and $p < 0.01$ levels, respectively, when compared with Group I.
##Significant difference at $p < 0.01$ level when compared with Group II.

significant findings. Alternatively, fetuses with external malformations and litters with viscerally malformed fetuses were not significantly different from controls. Visceral variations appeared in 5 of 52 Group I fetuses (9.6%), in 2 of 8 Group I litters (25.0%), in 13 of 24 Group II fetuses (54.2%), in 7 of 7 Group II litters (100.0%), in 9 of 24 Group III fetuses, in 6 of 6 Group III litters (100.0%), in 7 of 36 Group IV fetuses, and in 5 of 7 Group IV litters (71.4%). The characteristic findings of visceral variations included misshapen thymus, dilated ureter, misshapen hearts, dilated atria, and convoluted ureter.

The types and incidences of skeletal malformations, variations, and retardations are shown in Table 7. The fetal numbers with skeletal malformations in Group II were significantly higher when compared with the control group. Although fetal numbers with skeletal malformations in Group III were also slightly higher than controls, the differences were not statistically significant. Conversely, the fetal numbers with skeletal malformations in Group IV were significantly lower when compared with Group II. Skeletal malformations including fused sternums, cleaved sternums, fused ribs, and absent vertebra. Skeletal malformations appeared in 4 of 28 Group II fetuses (14.3%), in 2 of 7 Group II litters (28.6%), in 2 of 29 Group III fetuses (6.9%), and in 2 of

6 Group III litters (33.33%).

Significantly increased incidences of fetuses with skeletal variations and retardations were detected in all treatment groups when compared with controls. In contrast, no significant was detected in litters with affected fetuses in the treatment groups when compared with each group. Skeletal variations included bipartite ossification of the thoracic centrum, presence of cervical ribs, supernumerary lumbar vertebra, short or full supernumerary ribs, enlarged fontanels, and misaligned sternebrae. These variations occurred in 4 of 57 control fetuses (7.0%), in 2 of 8 control litters (37.5%), in 10 of 28 Group II fetuses (35.7%), in 7 of 7 Group II litters (100%), in 9 of 29 Group III fetuses (31%), in 5 of 6 Group III litters (83.3%), in 10 of 41 Group IV fetuses (24.4%), and in 4 of 7 group IV litters (57.1%). Skeletal retardations included dumbbell ossification of the thoracic centrum, incomplete ossification of supraoccipital areas, unossified pubis, incomplete ossification of parietal and interparietal areas. Skeletal retardations occurred in 7 of 57 control fetuses (12.3%), in 4 of 8 control litters (50%), in 24 of 28 Group II fetuses (85.7%), in 6 of 7 Group II litters (85.7%), in 21 of 29 Group III fetuses (72.4%), in 6 of 6 Group III litters (100%), in 20 of 41 Group IV fetuses (48.8%), and in 7 of 7 Group IV litters (100%). There was evidence of treatment-related reductions in fetal skeleton ossification. Group II rats demonstrated significant reductions in the ossification centers of the sternebrae, metacarpals, metatarsals, sacral vertebrae, and caudal vertebra. Group III rats had significant reductions in the sternebrae, metacarpals, and metatarsals, and Group IV significant reductions were seen in metacarpals and metatarsals.

## DISCUSSION

This study confirms and extends the findings of our previous study (Kim *et al.*, 2004) on 2-BP-induced developmental toxicity in rats and allow a better comprehension of the developmental phase specificity of 2-BP.

The 2-BP-induced maternal toxicities were represented increases in the incidence of clinical signs and reductions in body weight gain in the treatment groups. Treatment-related clinical signs were observed in all treatment groups, and included increased incidence and severity of reddish tears, nasal discharge, fur staining, weakness, piloerection, ataxia, and swelling at injection sites. The increased incidence of swelling at the injection sites could be attributed to the direct irritating effects of 2-BP injection, but the other clinical signs observed in the treatment groups were indicative of stress induction by the 2-BP. The significant reduction in maternal body weight gain observed in the late gestation periods was attributed to the test chemical administration and was consistent with the increased clinical signs in the groups. Increased post-implantation losses and decreased fetal and placental weights could have contributed to the suppressed body weight gain at term observed in the treatment groups. Accordingly, litter sizes and the fetal and placental weights of the groups were lower than in controls. Additionally, the clinical signs and body weight changes observed in the pregnant dams were similar among the treatment groups. These results suggested that neither the treatment period during pregnancy nor the metabolic activation induced by the PB pre-treatment affected the maternal effects of 2-BP injection on clinical signs and body weights in rats.

The developmental toxicity of 2-BP observed in the treatment groups included increases in fetal deaths and post-implantation loss, decreases in litter size and placental weight, increases in the incidence of fetal malformations, and fetal ossification delays. These results are consistent with our previous study which suggested that subcutaneous injection of 2-BP in rats on GD 6 through GD 19 resulted in various developmental toxicities at 1000 mg/kg (Kim *et al.*, 2004). However, the incidence and severity of developmental toxicities were slightly decreased when compared with the previous study, and this discrepancy is likely due to the shortening of the treatment duration. The potential adverse effects of 2-BP on embryo-fetal development seen in Group II were comparable to those seen in Group III, but were much higher than those in Group IV. This suggests that the metabolic activation induced by the PB pre-treatment did not affect the potential effects of 2-BP on embryo-fetal development in rats. In addition, these results indicate that the developmental toxic effects of 2-BP are much higher when administered during the first half of the organogenetic period.

Several teratogenicity studies investigated the potential effects of 2-BP on pregnant dams and embryo-fetal development (Ishikawa and Yamauchi, 2003; Kim *et al.*, 2003, 2004; Takeuchi *et al.*, 2004). Reproductive toxicity studies showed that the testicular or ovarian dysfunction induced by 2-BP treatment resulted from damaging the early types of spermatogenic cells in males or primordial follicles and their oocytes in females, indicating that highly proliferating cells/organs are primary targets of 2-BP (Omura *et al.*, 1999; Yu *et al.*, 1999). Ishikawa *et al.* (2001) showed an induction of micronuclei formation in preimplantation mouse embryos after maternal treatment with 2-BP accompanied by a decrease in embryo cell number. Recent *in vitro* studies also showed that 2-BP is an apparent DNA damaging agent (Wu *et al.*, 2002; Zhao *et al.*, 2002). The above results strongly suggest that the DNA damage by 2-BP might be involved in teratogenicity induced by 2-BP exposure in rat embryo-fetuses. It is apparent that 2-BP has a high potential for fetal-embryonic developmental toxicity when administered to pregnant females, although the teratogenic mechanism of 2-BP remains unclear. This study identified teratogenic characteristics of 2-BP and clarified the critical period of 2-BP associated embryotoxicity and teratogenicity in rats.

In conclusion, rats have greater susceptibility to 2-BP on embryo-fetal developmental toxicity in the first half of the organogenetic period rather than the second half. The metabolic activation induced by PB pre-treatment has little effect on the maternal and developmental toxicities of 2-BP in rats. Further studies are needed to clarify the mechanism of developmental toxicity of 2-BP.

## ACKNOWLEDGEMENTS

This work was supported by the Grant of the Korean Ministry of Education, Science and Technology (The Regional Core Research Program/Biohousing Research Institute).
